# Zhoushi Qi Ling decoction represses docetaxel resistance and glycolysis of castration-resistant prostate cancer via regulation of SNHG10/miR-1271-5p/TRIM66 axis

**DOI:** 10.18632/aging.203602

**Published:** 2021-10-06

**Authors:** Hongwen Cao, Dan Wang, Peng Sun, Lei Chen, Yigeng Feng, Renjie Gao

**Affiliations:** 1 Surgical Department I, Urology Department, Longhua Hospital Shanghai University of Traditional Chinese Medicine, Shanghai 200032, China

**Keywords:** prostate cancer, Zhoushi Qi Ling decoction, docetaxel resistance, Warburg effect, SNHG10/miR-1271-5p/TRIM66

## Abstract

Docetaxel resistance developed in half of castration-resistant prostate cancer (CRPC) patients hinders its long-term clinical application. The current study was designed to investigate the effects of Chinese medicine Zhoushi Qi Ling decoction on the docetaxel resistance of prostate cancer as well as elucidate the underlying molecular mechanism. In our study, Qi Ling significantly decreased viability and colony formation as well as increased apoptosis of docetaxel-resistant (DR) CRPC cells. Qi Ling-treated DR cells exhibited decreased glucose consumption, lactate release and pyruvate production. Moreover, lncRNA SNHG10 was upregulated in DR tissues of CRPC patients and was negatively correlated with the progression-free survival. Bioinformatics analysis indicated miR-1271-5p as the associated miRNA possibly binding with SNHG10. miR-1271-5p up-regulation dramatically decreased the luciferase activity of SNHG10 in DR cells. SNHG10 knockdown sharply increased the expression of miR1271-5p in DR cells. Targetscan predicted TRIM66 as one of the downstream targets of miR-1271-5p. miR-1271-5p up-regulation drastically reduced luciferase activity as well as TRIM66 expression in DR cells. Also, the knockdown of SNHG10 remarkably suppressed the expression of TRIM66 in DR cells. Additionally, Qi Ling treatment reduced SNHG10 and TRIM66, while increased miR1271-5p, in DR cells. In summary, Qi Ling inhibited docetaxel resistance and glycolysis of CRPC possibly via SNHG10/miR-1271-5p/TRIM66 pathway.

## INTRODUCTION

Despite dramatic progress in therapeutics having been made in recent years, prostate cancer remains one of the leading causes of cancer-related death in American men [1]. As prostate cancer progression is androgen-dependent during the early stage [2], androgen deprivation therapy (ADT) is the best choice for treatment of advanced prostate cancer for a short time. While 50% of these ADT-treated patients can develop resistance to ADT, which eventually results in metastatic castration-resistant prostate cancer (CRPC). Nowadays, docetaxel is the first-line chemotherapeutic drug for patients with metastatic CRPC, while the docetaxel resistance induced by long-term usage has hindered its clinical application [3, 4]. Hence, it is in great need to elucidate the mechanism of docetaxel resistance and develop effective medicine to eliminate docetaxel resistance in CRPC patients.

The dysfunction in energy metabolism usually occurs in malignant tumors [5]. In physical condition, the cells could metabolize glucose via oxidative phosphorylation. Instead, cancer cells prefer to obtain energy via transferring glucose into lactate (aerobic glycolysis). This is a phenomenon termed “Warburg effect” which is essential to proliferation of cancer cells and patients’ prognosis prediction [6, 7]. This effect also appears in prostate cancer cells [8–10].

Long non-coding RNAs (lncRNAs) have long been recognized as transcription by-products of RNA polymerase II with no biological functions [11, 12]. In recent years, lncRNAs have been presented as critical regulators of differentiation, proliferation, apoptosis, and metastasis during cancer pathogenesis [13–15]. LncRNAs as well as other non-coding RNAs have emerged as promising molecular targets for tumor diagnosis and therapy. LncRNA SNHG10 is reported to promote glucose uptake via interacting with related miRNA in osteosarcoma [16]. It is a pity that the biological role of SNHG10 in glucose metabolism in the cancer remains unclear. Our group previously found that Chinese medicine Zhoushi Qi Ling decoction suppressed the development of prostate cancer by negatively regulating TRIM66/HP1γ/AR axis [17]. The present study aimed to investigate whether Qi Ling affects docetaxel resistance and aerobic glycolysis as well as the underlying mechanisms.

## MATERIALS AND METHODS

### Qi Ling

“Qi Ling” was composed of kushan (150 g), rubescens (300 g), raw astragalus (150 g), turmeric (90 g), psoraleae (150 g), cooked rehmannia glutinosa (150 g), motherwort (150 g), and processed licorice (90 g). It was boiled in hot water for 3–4 hours, then refilled to 1 L.

### Cell culture

PC3 and DU145, as two human prostate cell lines, were purchased from American Type Culture Collection (Manassas, VA, USA). The cells were maintained in RPMI-1640 medium (Gibco, Grand Island, NY) containing 10% fetal bovine serum (Gibco) and 1% penicillin/streptomycin (Gibco) at 37°C with 5% CO_2_. Docetaxel-resistant (DR) cell lines (PC3-DR and DU145-DR) were successfully established via exposing monolayer-cultured cells to incremental concentrations of docetaxel (dissolved in DMSO; Sigma, St. Louis, MO, USA) in scheduled procedures according to the methods described previously [18, 19]. The established DR cells were regularly cultured in the medium containing docetaxel (10 nM).

### Clinical samples

Human study was approved by LONGHUA Hospital Shanghai University of Traditional Chinese Medicine. All patients signed consent before surgery. Briefly, 56 docetaxel-free (DR-free) and 44 DR tissues were collected from prostate cancer patients during prostatectomy.

### Cell survival assay

Cell survival was determined by colony formation and Cell-counting Kit-8 (CCK-8) assays. CCK-8 assay was conducted using the corresponding detection Kit (Beyotime, Haimen, China) according to the manufacturer’s instruction. In brief, equal amounts of DR cells were seeded 24 h before 48-h docetaxel treatment at concentrations from 5 to 400 nM. CCK-8 reagent was added, incubated for 3 h to measure the absorbance at 450 nm. For colony formation assay, 1000 cells per well (in 6-well plate) were seeded 24 h before treated with 10 nM docetaxel. After 10 days of culture, the cells were washed using sterile phosphate-buffered saline and fixed using 4% paraformaldehyde followed by staining with crystal violet. The colonies formed in each group were counted manually.

### Quantitative real-time polymerase chain reaction (qRT-PCR)

Total RNAs were extracted using TriZol reagent (Invitrogen, Waltham, MA, USA). Transcription of cDNA was conducted using PrimeScript RT reagent (TaKaRa, Dalian, China). qRT-PCR was performed using SYBR Green PCR Kit (TaKaRa) on ABI 7300 system (Applied Biosystems, Waltham MA, USA). Primers were used as follows (5′ to 3′): *SLC2A1*, ATTGGCTCCGGTATCGTCAAC (Forward), GCTCAGATAGGACATCCAGGGTA (Reverse); *PFKP*, CGCCTACCTCAACGTGGTG (Forward), ACCTCCAGAACGAAGGTCCTC (Reverse); *PKM*, ATGTCGAAGCCCCATAGTGAA (Forward), TGGGTGGTGAATCAATGTCCA (Reverse); *LDHA*, ATGGCAACTCTAAAGGATCAGC (Forward), CCAACCCCAACAACTGTAATCT (Reverse); *SNHG10*, CCAGCTTAGATTCATTGATTCC (Forward), TTAAGTGCACCAGATGCTG (Reverse); *TRIM66*, GCCCTCTGTGCTACTTACTC (Forward), GCTGGTTGTGGGTTACTCTC (Reverse); miR-1271-5p, CAGCACTTGGCACCTAGCA (Forward), TATGGTTGTTCTCCTCTCTGTCTC (Reverse); *U6*, GCTTCGGCAGCACATATACTAAAAT (Forward), CGCTTCACGAATTTGCGTGTCAT (Reverse); *β-actin*, CGTCATACTCCTGCTTGCTG (Forward), GTACGCCAACACAGTGCTG (Reverse). The relative expression of target genes was analyzed using the 2^−ΔΔCt^ method with *U6* as the internal control for miR-1271-5p and *β-actin* for the other genes.

### Western blot

Cells of different groups were collected, then lysed using radioimmunoprecipitation buffer containing protease inhibitor. Equal amounts of proteins were loaded and separated using sodium dodecyl sulfate–polyacrylamide gel electrophoresis gel (10%) followed by being transferred to polyvinylidene fluoride membranes (Bio-Rad, Hercules, CA, USA). After blocking with bovine serum albumin (5%) at room temperature for 2 h, membranes were then incubated with corresponding primary antibodies probing TRIM66 (1:1300; Abcam, Cambridge, MA, USA) and β-actin (1:2400; Santa Cruz, CA, USA) at 4°C overnight. After incubation with corresponding horse radish peroxidase-conjugated secondary antibodies (1:2500; Santa Cruz, Dallas, TX, USA) at room temperature for 2 h, the blots were visualized using an ECL kit (Thermo Fisher, Waltham, MA, USA).

### Cell apoptosis

Cell apoptosis was evaluated via detecting DNA fragmentation and caspase-3 activity. Caspase 3 activity was determined using a commercial caspase 3/7 assay kit (Promega, Madison, WI, USA). Luminescence was detected using a microplate reader (Berthold, Calmbacher, Germany). DNA fragmentation assay was conducted via assessing the levels of cytoplasmic histone-associated DNA fragment in each group using an ELISA kit for cell death detection (Roche, Mannheim, Germany) according to the recommended instructions.

### Assays of glucose consumption, pyruvate and lactate production

Cell numbers from different groups were determined before detection. Cells were collected to analyze the pyruvate level using a commercial detection kit (Sigma). Culture medium was harvested and the lactate level as well as glucose after different treatments were measured. The lactate production or glucose consumption was normalized using cell number determined before detection.

### Cell transfection

In order to knock down SNHG10 expression, cells were transiently transfected with two SNHG10-pecific siRNAs, si-SNHG10-1 (GCAACCGCTTTGTTAGTTAAT) and si-SNHG10-2 (GCGCGCGATTATTTCTCTAGA), or negative control, (si-NC, ACATGACTGTATCTGTCTAGT), using Lipofectamine 3000 (Invitrogen). SNHG10 was overexpressed via transfecting pcNDA3.1/SNHG10 vectors conducted through inserting cDNA of SNHG10 into pcDNA3.1 vectors as previous described [20]. Commercial miR-1271-5p mimics and negative control (miR-NC) were provided by GeneWiz (Suzhou, China). Cell lysates were prepared 48 h after transfection for further assays.

### RNA pull down assay

Lysates collected from cells with SNHG10-knockdown were incubated with biotinylated RNA probes. The miR-1271-5p sequence were biotinylated and a nonsense RNA sequence with biotin label served as the negative control. The enrichment of targeted RNA was determined by RT-qPCR.

### Luciferase reporter assay

The luciferase reporter plasmid was constructed via inserting different wild-type (WT) or mutated (MUT) sequences. The target sequences were as follows: SNHG10-WT (Forward) CCTTCTCGAGAGCCTCATCCTACTGCCTT, (Reverse) CCTTGCGGCCGCATTTTGGAAAGAGTTTAA; SNHG10-MUT (Forward) CTGAGCAGCCGGCGCGCGATCGCC, (Reverse) GGCGATCGCGCGCCGGCTGCTCAG; TRIM66-WT, (Forward) CCTTCTCGAGGAGCCAAAAGGAGACTGGGC, (Reverse) CCTTGCGGCCGCTTCTTGGTAAAGAAAAGTGCTGTT; TRIM66-Mut (Forward) CAGTGACTGCCCAGTTCCAACAT, (Reverse) ATGTTGGAACTGGGCAGTCACTG. The above plasmids were co-transfected with negative control or miR-1271-5p mimics, respectively. The luciferase activity was measured using the luciferase reporter assay (Promega, Madison, WI, USA).

### Statistical analyses

Data were presented as mean ± SD. Experiments were repeated in triplicate unless specified. Statistical analysis was carried out using SPSS 16.0 and differences between indicated groups were compared using Student’s *t*-test, One-way, Two-way ANOVA with appropriate post hoc test. It was considered statistically significant when *p* value was less than 0.05.

## RESULTS

### Qi Ling impaired docetaxel resistance

**Figure 1:**
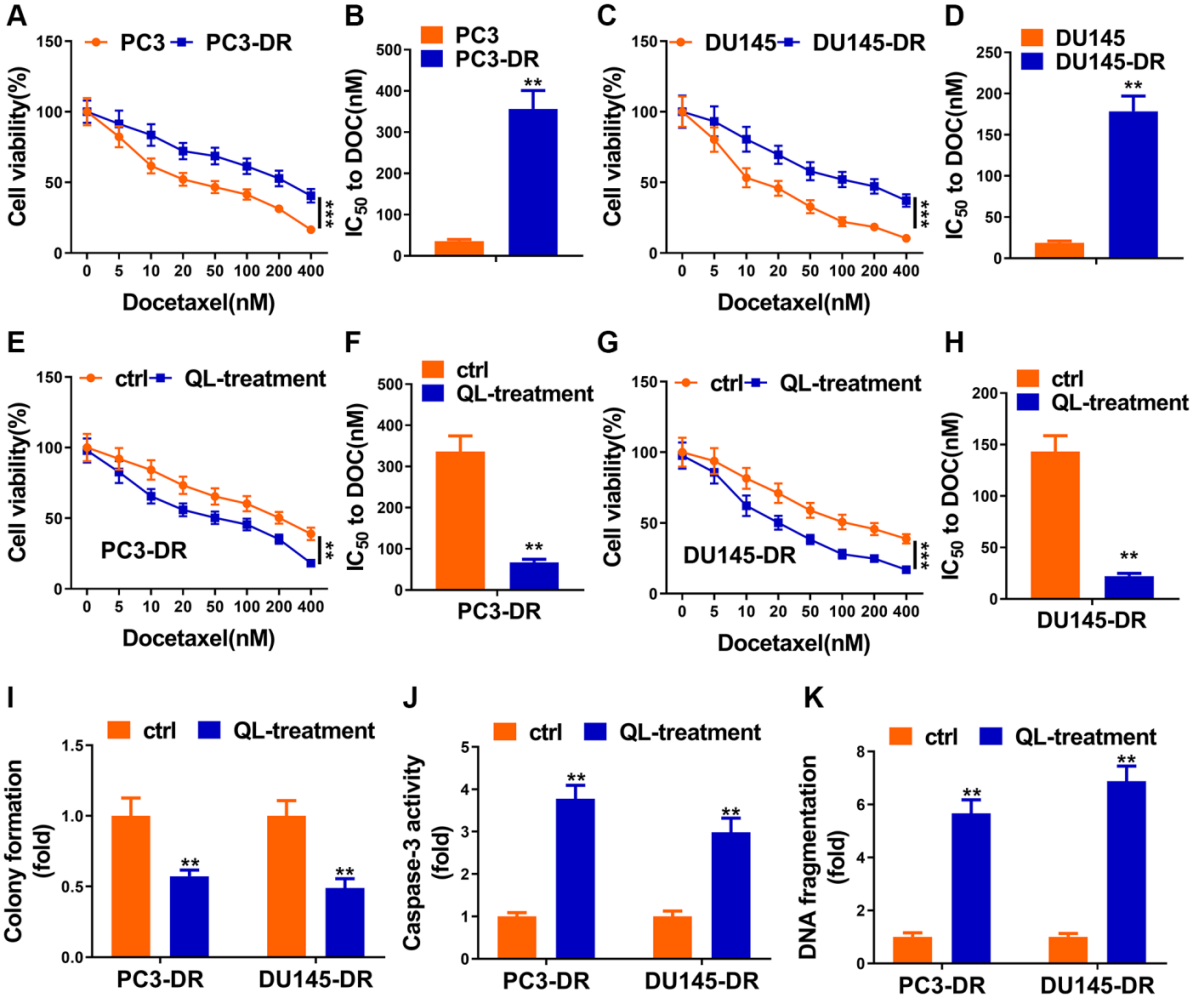
**Qi Ling impaired docetaxel resistance of CRPC cells.** (**A** and **B**) Parental PC3 cells and the DOC-resistant counterpart (PC3-DR) were subjected to indicated concentrations of docetaxel (0–400 nM) for 48 h, cell viability and IC50 value of docetaxel were determined by CCK-8 assay. (**C** and **D**) Parental DU145 cells and the DOC-resistant counterpart (DU145-DR) were subjected to the indicated concentrations of docetaxel (0–400 nM) for 48 h, cell viability and IC50 value of docetaxel were determined by CCK-8 assay. (**E** and **F**) PC3-DR cells was cultured in normal media (ctrl) or media supplement with Qi Ling (QL-treatment) with the treatment of the indicated concentrations of docetaxel (0–400 nM) for 48 h, cell viability and IC50 value of docetaxel were determined by CCK-8 assay. (**G** and **H**) DU145-DR cells was cultured in normal media (ctrl) or media supplement with Qi Ling (QL-treatment) with the treatment of the indicated concentrations of docetaxel (0–400 nM) for 48 h, cell viability and IC50 value of docetaxel were determined by CCK-8 assay. (**I**) Colony formation assay showed cell viabilities of PC3-DR and DU145-DR cells cultured in normal media (ctrl) or media supplement with Qi Ling (QL-treatment) with the treatment of docetaxel (10 nM). (**J** and **K**) Cell apoptosis of PC3-DR and DU145-DR cells cultured in normal media (ctrl) or media supplement with Qi Ling (QL-treatment) with the treatment of docetaxel (10 nM) was measured by DNA fragmentation and Caspase-3 activity assays. Values are mean ± SD. ^**^P < 0.01; ^***^P < 0.001.

As shown in Figure 1A and 1B, PC3-DR cells showed higher cell viability 48 h after treatment with indicated docetaxel, and IC50 value of this cell line to docetaxel was obviously higher than that of control. Another cell line, DU145-DR, performed similarly with that of PC3-DR (Figure 1C and 1D). After the successful construction of docetaxel-resistant cell lines, we next applied Qi Ling treatment to see whether their responses to docetaxel would change. Cell viability of Qi ling-treated docetaxel-resistant cell lines was significantly reduced in the same scenario of docetaxel treatment as evidenced in Figure 1E and 1G. The IC50 values of DR cell lines to docetaxel were remarkably decreased after Qi Ling treatment (Figure 1F and 1H). Colonies of docetaxel-resistant cells in Qi Ling treated group were also obviously decreased in the context of low-dose docetaxel compared with the untreated control cells, co-suggesting that Qi Ling suppressed proliferation of docetaxel-resistant CRPC cells when treated with docetaxel (Figure 1I). Caspase 3 activity and DNA fragmentation assays showed evident apoptotic levels of Qi Ling-treated docetaxel-resistant cells when treated with docetaxel (Figure 1J and 1K). In summary, Qi Ling-treated docetaxel-resistant CRPC cells showed decreased proliferation and increased apoptosis in the scenario of docetaxel treatment, indicating that Qi Ling enhanced the sensitivity of CRPC cells to docetaxel.

### Qi Ling regulated the Warburg effect of CRPC cells

**Figure 2:**
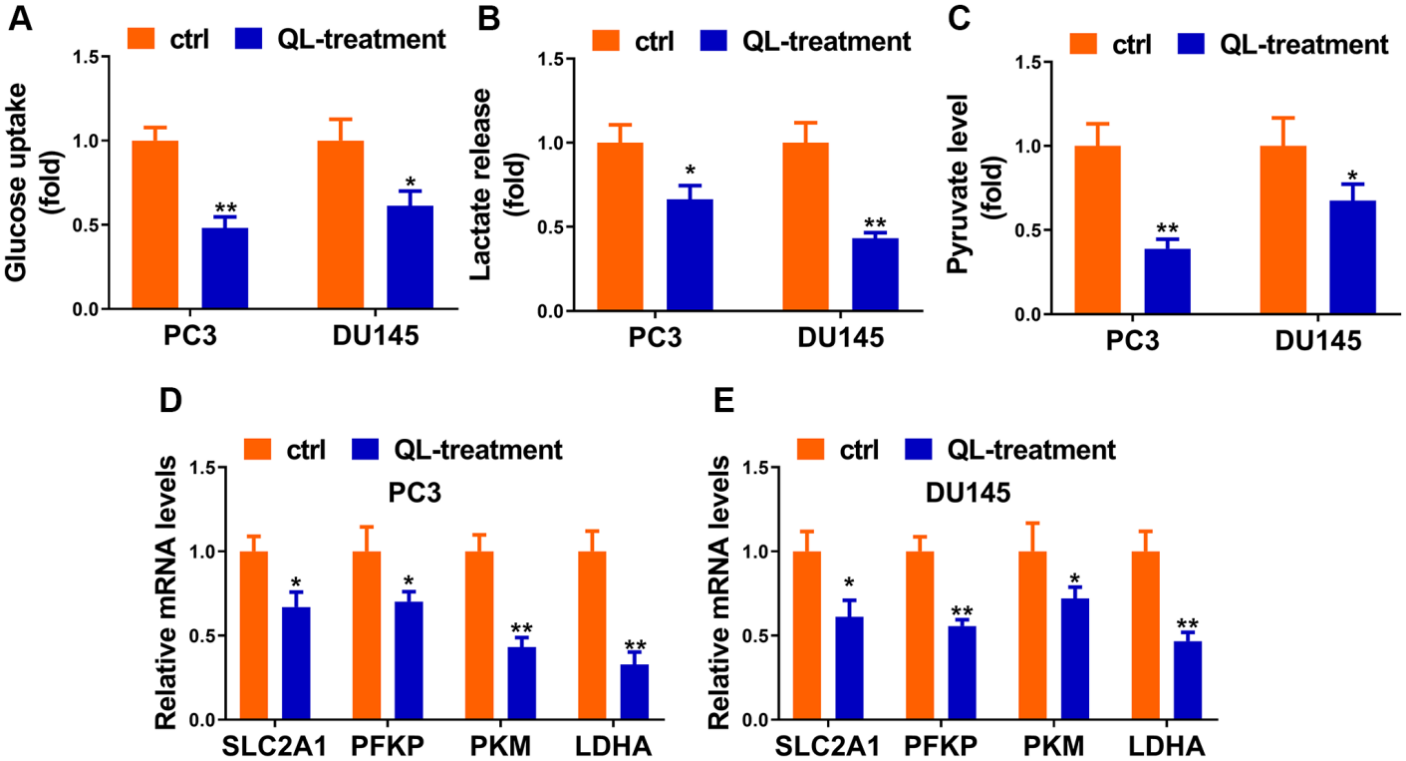
**Qi Ling regulated the Warburg effect of CRPC cells.** Relative glucose consumption (**A**), pyruvate concentration (**B**), and lactate production (**C**) were assessed in PC3-DR and DU145-DR cells. (**D** and **E**) mRNA levels of glycolytic components (SLC2A1, PFKP, PKM and LDHA) in PC3-DR and DU145-DR cells were examined by qRT-PCR. Values are mean ± SD. ^*^P < 0.05; ^**^P < 0.01.

Considering Warburg effect is essential phenomena during the development of tumorigenesis, we hypothesized that Qi Ling might affect the glycolysis of prostate cancer cells. As expected, Qi Ling treatment remarkably reduced glucose consumption, lactate release and pyruvate production of CRPC cells compared to control (Figure 2A–2C). Meanwhile, the expression of glycolysis-related genes, SLC2A1, PFKP, PKM, and LDHA, presented in a pronounced drop in Qi Ling-treated group compared with the control group (Figure 2D and 2E). These findings meet our expectation that Qi Ling regulated the Warburg effect of CRPC cells.

### SNHG10 was inhibited by Qi Ling and associated with docetaxel resistance of CRPC

**Figure 3:**
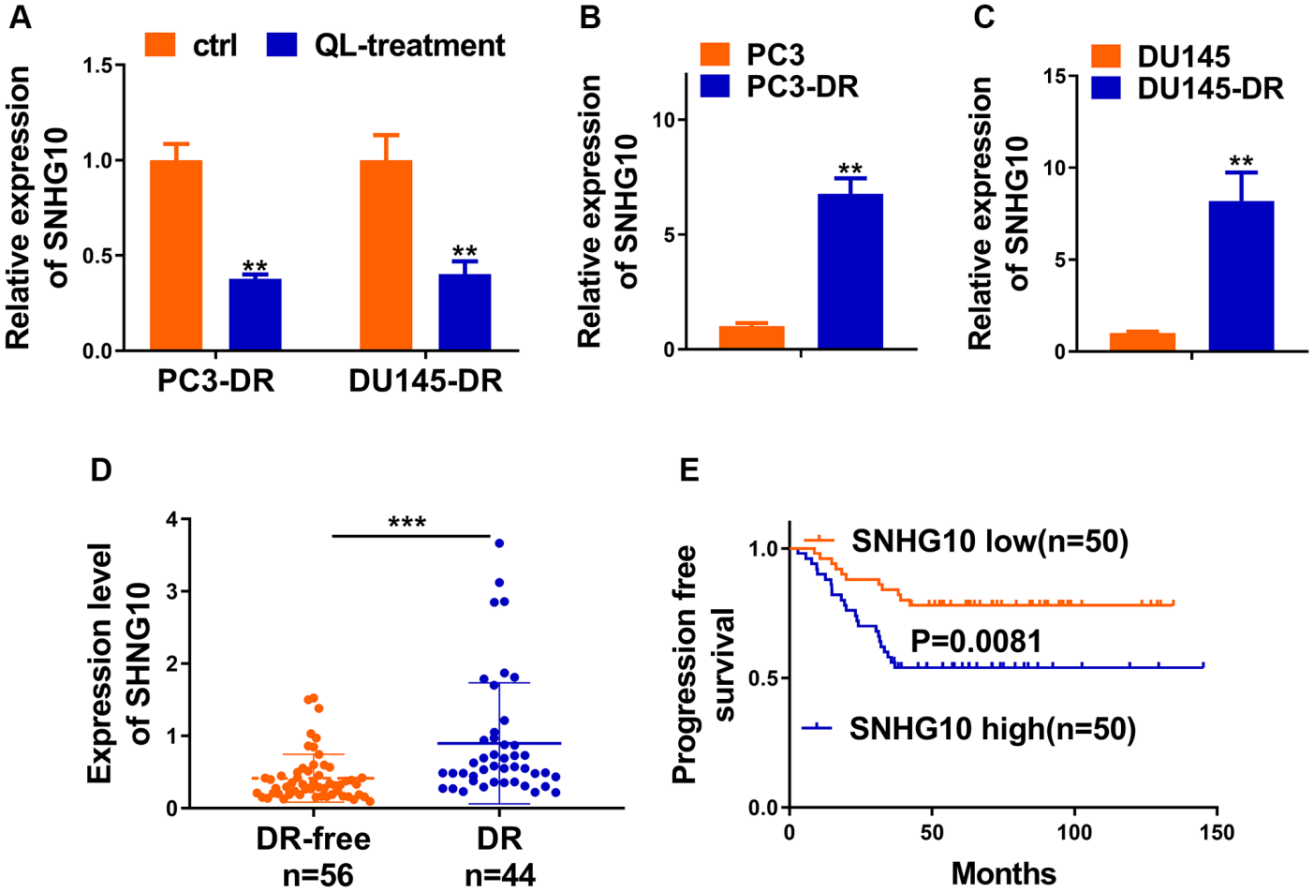
**SNHG10 was inhibited by Qi Ling and associated with docetaxel resistance of CRPC.** (**A**) RNA level of SNHG10 in PC3-DR and DU145-DR cells was examined by qRT-PCR. (**B** and **C**) qRT-PCR was performed to detect the expression level of SNHG10 in PC3 and DU145 cells or their DOC-resistant counterparts (PC3-DR and DU145-DR). (**D**) RNA level of SNHG10 in 56 docetaxel-free (DR-free) and 44 DR tissues of CRPC patients was examined by qRT-PCR. (**E**) Kaplan-Meier analysis of the correlation between SNHG10 expression and progression-free survival of CRPC patients. ^*^P < 0.05; ^**^P < 0.01; ^***^P < 0.001.

It was worth noting that increased glucose uptake and cell proliferation in osteosarcoma were correlated with enhanced lncRNA SNHG10 in a recent report [16]. We first explored whether Qi Ling could affect the expression of SNHG10 in CRPC cells. Figure 3A indicated that SNHG10 exhibited sharp decreases after Qi Ling treatment in both DR cell lines, suggesting that Qi Ling remarkably rectifies the abnormal increase of SNHG10 expression *in vitro*. Results of *in vitro* assays showed that both DR cell lines presented obvious enhanced mRNA expression of SNHG10 compared with the corresponding counterpart cell lines (Figure 3B and 3C). We then hypothesized that SNHG10 might be correlated with drug resistance of CRPC cells. We analyzed the difference of SNHG10 in DR-free and DR tissues of clinically diagnosed patients with prostate cancer, which evidenced a significant increase in DR tissues in comparison with that in DR-free tissues (Figure 3D). Correlation analysis presented the negative correlation between progression-free survival and SNHG10 expression of CRPC patients (Figure 3E). The clinical and *in vitro* findings co-suggest that SNHG10 might correlate with docetaxel resistance of CRPC cells as its abnormal increase in DR tissues and DR cell lines.

### SNHG10 up-regulated TRIM66 via sponging miR-1271-5p

**Figure 4:**
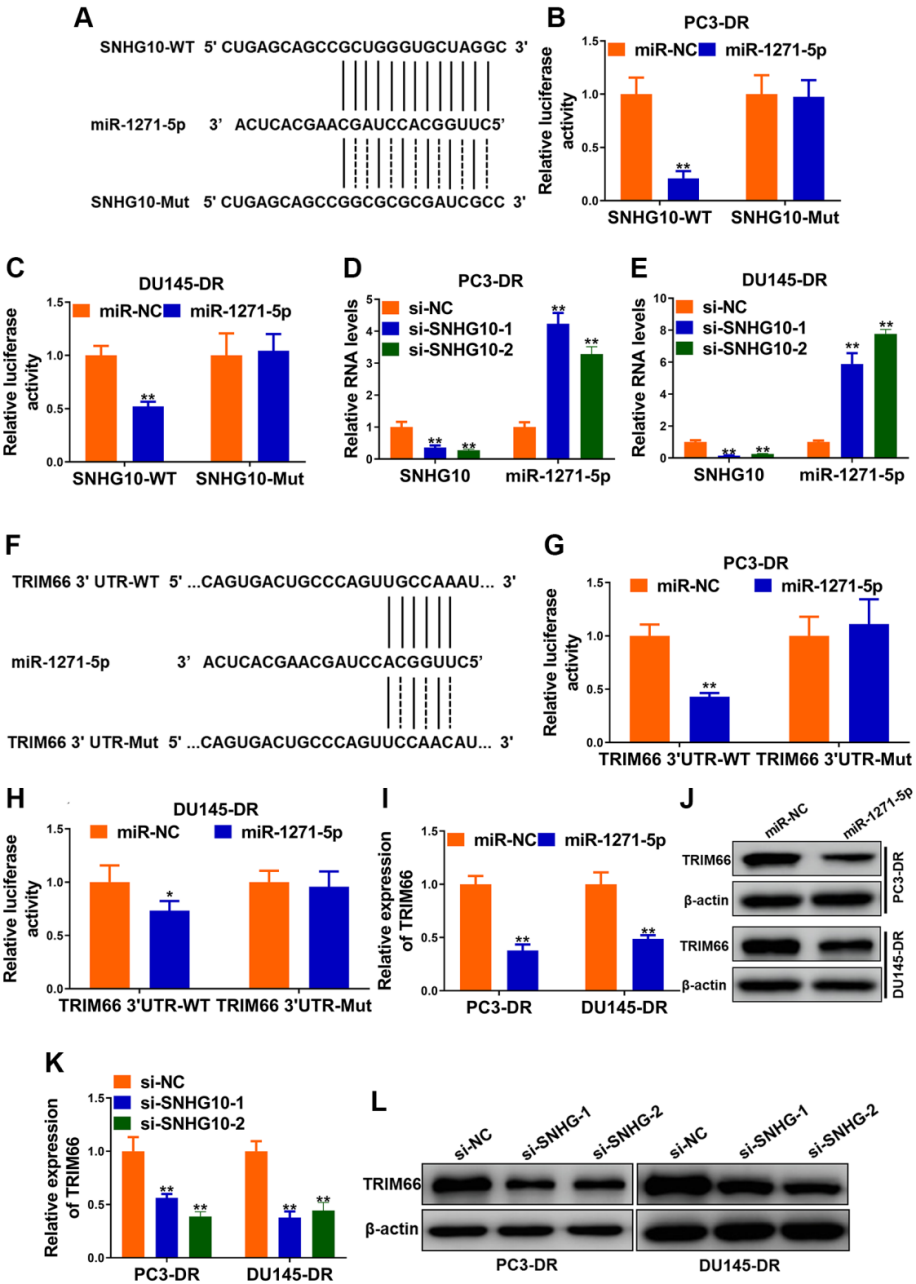
**SNHG10 up-regulated TRIM66 via sponging miR-1271-5p.** (**A**) miR-1271-5p binding sites in SNHG10 and site mutagenesis. (**B** and **C**) Luciferase activity in PC3-DR and DU145-DR cells. (**D** and **E**) RNA levels of SNHG10 and miR-1271-5p in PC3-DR and DU145-DR cells transfected with siRNAs against SNHG10 (si-SNHG10-1 and si-SNHG10-2) or negative control siRNAs (si-NC) were detected by qRT-PCR. (**F**) miR-1271-5p binding sites in TRIM66 3’UTR and site mutagenesis. (**G** and **H**) Relative luciferase activity in PC3-DR and DU145-DR cells. (**I** and **J**) mRNA and protein level of TRIM66 in PC3-DR and DU145-DR cells transfected with miR-1271-5p mimics or mimics negative control (miR-NC) were determined by qRT-PCR and Western blot. (**K** and **L**) mRNA and protein level of TRIM66 in PC3-DR and DU145-DR cells transfected with siRNAs against SNHG10 (si-SNHG10-1 and si-SNHG10-2) or negative control siRNAs (si-NC) were determined by qRT-PCR and Western blot. Values are mean ± SD. ^*^P < 0.05; ^**^P < 0.01.

Bioinformatics analysis was conducted to explore the downstream genes of SNHG10. miRcode (http://www.mircode.org/mircode/) was firstly applied and miR-1271-5p was predicted to be associated miRNAs possibly binding with SNHG10. Another online software (https://bibiserv.cebitec.uni-bielefeld.de/rnahybrid) was employed to predict sites possibly binding with SNHG10. Site mutagenesis was also performed (Figure 4A). Next, miR-1271-5p transfection significantly reduced the luciferase activity of DR cells carrying SNHG10-WT, while no obvious changes were observed when DR cells carrying SNHG10-Mut (Figure 4B and 4C), demonstrating the combination between SNHG10 and miR-1271-5p. SNHG10 knockdown sharply increased the expression of miR1271-5p in DR cells, suggesting that SNHG10 negatively regulates miR-1271-5p via binding in CRPC cells (Figure 4D and 4E). The downstream targets of miR-1271-5p were predicted using Target Scan, and one of genes we particularly have interest in, TRIM66, was in them (Figure 4F). Luciferase reporter assay manifested that miR-1271-5p transfection drastically reduced luciferase activity of TRIM66-WT group while not TRIM66-MUT group in PC3-DR and DU145-DR cells, revealing the combination between miR-1271-5p and TRIM66 (Figure 4G and 4H). Additionally, miR-1271-5p transfection significantly decreased both of the mRNA and protein expression of TRIM66 in PC3-DR and DU145-DR cells, demonstrating the negative regulation of TRIM66 by miR-1271-5p (Figure 4I and 4J). The knockdown of SNHG10 remarkably reduced the expression of TRIM66 in both DR cell lines (Figure 4K and 4L), suggesting the positive regulation of TRIM66 by SNHG10 in CRPC cells. Accumulatively, SNHG10 upregulated TRIM66 expression via sponging miR-1271-5p to block its negative control on TRIM66, which was possible molecular mechanism underlying the docetaxel resistance of CRPC cells.

### Qi Ling inhibited docetaxel resistance and glycolysis through SNHG10/miR-1271-5p/TRIM66 axis in CRPC cells

**Figure 5:**
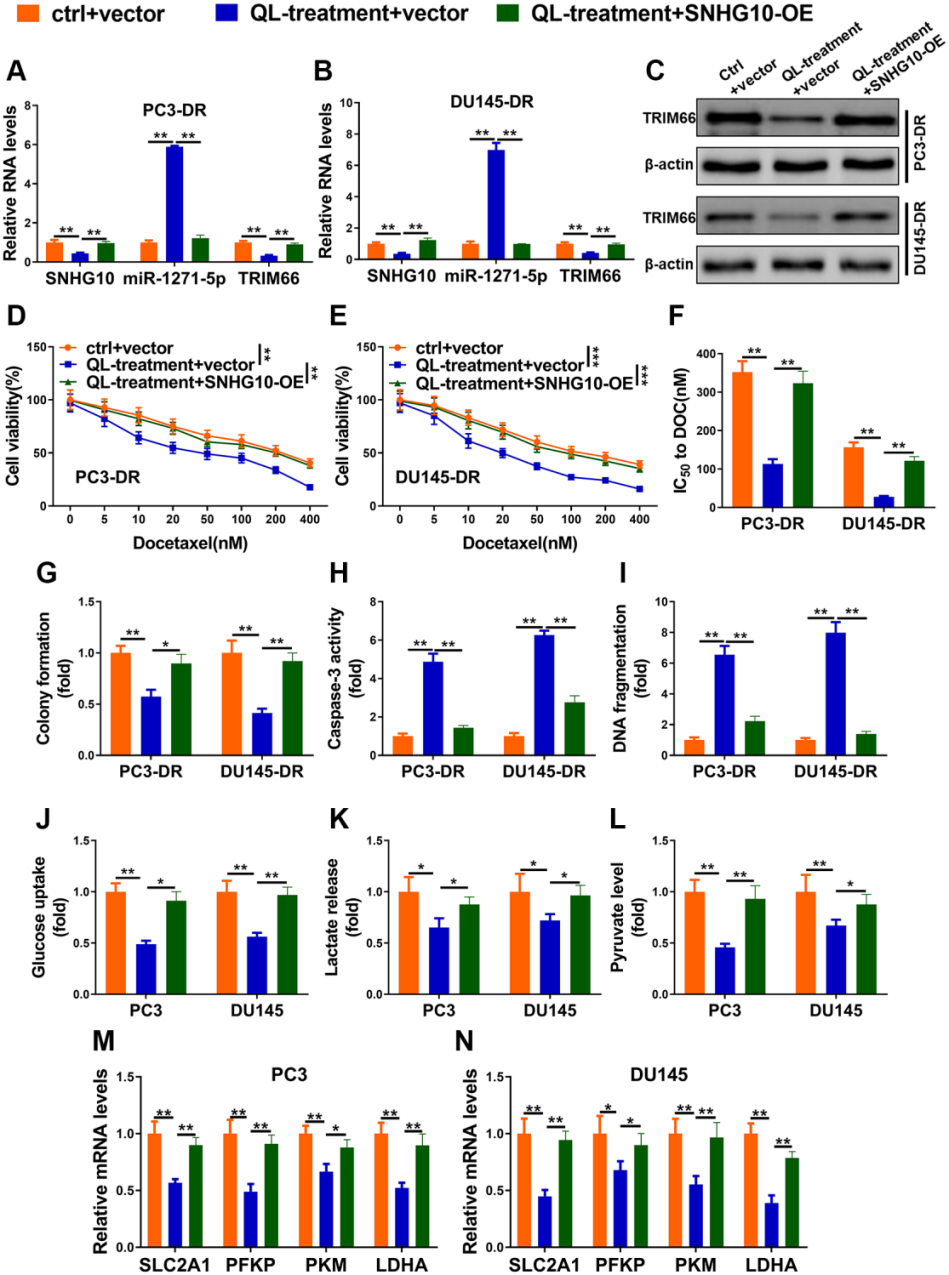
**Qi Ling inhibited docetaxel resistance and glycolysis through SNHG10/miR-1271-5p/TRIM66 axis in CRPC cells.** (**A**–**I**) PC3-DR and DU145-DR cells were transfected with empty vector and cultured in normal media (ctrl + vector), transfected with empty vector and cultured in media supplement with Qi Ling (QL-treatment + vector) or transfected with SNHG10 overexpression plasmid and cultured in media supplement with Qi Ling (QL-treatment + SNHG10-OE). (**A** and **B**) RNA levels of SNHG10, miR-1271-5p and TRIM66 were examined by qRT-PCR. (**C**) Protein level of TRIM66 was determined by Western blot. (**D**–**G**) Cell viability and IC50 value of docetaxel were determined by CCK-8 assay and colony formation assay. (**H** and **I**) Cell apoptosis was measured by Caspase-3 activity assay and DNA fragmentation assay. (**J**–**N**) PC3 and DU145 cells were transfected with empty vector and cultured in normal media (ctrl + vector), transfected with empty vector and cultured in media supplement with Qi Ling (QL-treatment + vector) or transfected with SNHG10 overexpression plasmid and cultured in media supplement with Qi Ling (QL-treatment + SNHG10-OE). Relative glucose consumption (**J**), pyruvate concentration (**K**), and lactate production (**L**) were assessed in PC3 and DU145 cells (**M** and **N**) mRNA levels of glycolytic components (SLC2A1, PFKP, PKM and LDHA) in PC3 and DU145 cells were examined by qRT-PCR. Values are mean ± SD. ^*^P < 0.05; ^**^P < 0.01; ^***^P < 0.001.

Two DR cell lines were divided into control + vector (ctrl + vector), Qi Ling treatment + vector (QL-treatment + vector), and QL-treatment + SNHG10-OE groups, respectively. Qi Ling treatment significantly reduced the expression of SNHG10 and TRIM66 meanwhile increased miR-1271-5p in DR cells (Figure 5A and 5B). Whereas overexpression of SNHG10 remarkably rectified the changes of the above three factors triggered by Qi Ling treatment in DR cells further verifying the upstream position of SNHG10 of this axis. These findings demonstrate that Qi Ling affects the expression of SNHG10/miR-1271-5p/TRIM66 axis in DR cells and SNHG10 overexpression could correct the influence of Qi Ling on this axis. The protein level of the downstream factor TRIM66 of this axis changed in consistent with the mRNA expression profile of each group (Figure 5C). Overexpression of SNHG10 also adjusted the decreased cell viability, IC50 to DOC, as well as colony formation induced by Qi Ling treatment in DR cells to the control level (Figure 5D–5G), which suggests that SNHG10 played a critical role in the suppression of Qi Ling to CRPC cells. Figure 5H and 5I showed decreased caspase-3 activity and DNA fragmentation level in DR cells of QL-treatment + SNHG10-OE group, indicating SNHG10 is critic in the apoptosis promotion of Qi Ling to CRPC cells. The disturbed glucose uptake, lactate release, pyruvate production, as well as reduced expression of glycolysis-related genes in Qi Ling-treated DR cells were also modified almost to the control level via SNHG10 overexpression (Figure 5J–5N). Combined with the above described findings that SNHG10 acts as the upstream factor and reduces TRIM66 via sponging miR-1271-5p, it is concluded that Qi Ling suppressed docetaxel resistance and glycolysis through SNHG10/miR-1271-5p/TRIM66 axis in CRPC cells.

## DISCUSSION

In this study, we found that Qi Ling impaired the docetaxel resistance and regulated the Warburg effect of CRPC cells. LncRNA SNHG10 was upregulated in DR tissues and was negatively correlated with survival of patients. SNHG10 up-regulated TRIM66 via sponging miR-1271-5p, which might be the molecular basis of the docetaxel resistance of CRPC cells. Finally, we found that Qi Ling might inhibited docetaxel resistance and glycolysis of CRPC via SNHG10/miR-1271-5p/TRIM66 axis.

Chemotherapeutic resistance is one of the major concerns when clinicians make therapeutic strategies for advanced cancer patients and can eventually result in cancer progression as well as poor prognosis [21]. In recent years, compounds extracted from traditional Chinese medicines have been reported to be a neoadjuvant to reduce or even reverse drug resistance via inhibiting proliferation, promoting apoptosis, as well as regulating metabolism of cancer cells [22]. Zou J et al. pointed that ginsenoside Rg3 inhibited the growth and promoted cell apoptosis of gemcitabine-resistant pancreatic cancer cells [23]. Yang L et al. demonstrated that Oblongifolin C impaired gemcitabine-induced drug resistance in pancreatic cancer through down-regulation of Src [24]. Wang X et al. evidenced the reversal of gemcitabine resistance by melittin in pancreatic ductal adenocarcinoma cells via downregulating cholesterol pathway [25]. A recent study pointed that Fuzheng Yiliu decoction enhanced the anti-cancer efficacy of docetaxel in a CRPC murine model [26]. Similarly, we found that Qi Ling decoction significantly impaired the docetaxel resistance of CRPC cells *in vitro*.

Warburg effect, characterized by enhanced aerobic glycolysis, is the important biochemical feature of cancer cells, meaning the glucose dependence of cancer cells [27]. Warburg effect is related with tumorigenesis and plays critic roles in the development of chemotherapeutic resistance [28]. Many traditional Chinese medicines have been reported to re-sensitize drug-resistant cancer cells to chemotherapeutic reagents. Baicalein, a flavonoid extracted from *Scutellaria baicalensis*, is reported to reverse hypoxia-induced 5-fluorouracil resistance, via inhibiting glycolysis of gastric cancer cells [29]. In the present study, we found Qi Ling decoction significantly inhibited Warburg effect of CRPC cells, which could reasonably relate the reverse of docetaxel resistance by Qi Ling with reduced Warburg effect.

SNHG10 functions as an oncogenic lncRNA overexpressed in liver cancer and positively regulates carcinogenesis and metastasis [30]. SNHG10 was reported to be involved in the glucose metabolism in osteosarcoma via increasing the methylation of miR-218 [16]. However, the role of SNHG10 in prostate cancer hasn’t been mentioned. In the present study, we found SNHG10 is upregulated in CRPC tissues with docetaxel resistance and correlated with low survival rate which enriched the finding that upregulated SNHG10 predicted poor prognosis [30]. Bioinformatics analysis raised the candidate miRNA associated with SNHG10, that is miR-1271-5p, also participates the progression of prostate cancer after being sponged by circular RNA circMBOAT2 [31]. We previously evidenced that TRIM66 promoted malignant behaviors of prostate cancer cells, including proliferation, migration, and invasion [32]. Recently, we also described the negative regulation of TRIM66/HP1γ/AR axis by Qi Ling [17]. The present study re-validated the negative control of TRIM66 by Qi Ling on one hand and further explored the upstream factors of TRIM66 regulated by Qi Ling. The findings in our study haven’t been verified *in vivo*, and we will perform animal experiment to make our findings more solid in the future.

## CONCLUSIONS

In summary, Qi Ling inhibited docetaxel resistance and glycolysis of CRPC via SNHG10/miR-1271-5p/TRIM66 pathway. This study first revealed the inhibition of Qi Ling on docetaxel resistance and glycolysis of CRPC cells. In addition, this study revealed anti-tumor activity of Qi Ling against prostate cancer, providing experimental evidences for chemotherapy and chemo-sensitivity study of other Chinese medicines.
